# Assessment of Serum Calcium, Total Alkaline Phosphatase, Vitamin D Levels, and Quality of Life in Patients on Prolonged Antiepileptic Therapy: A Cross-Sectional Study

**DOI:** 10.7759/cureus.94024

**Published:** 2025-10-07

**Authors:** Sharath Nallaperumal, Padma V, Vinatha M.C, Lakshmi Chaitanya Varma Pusapati, Ishaivanan M

**Affiliations:** 1 Department of General Medicine, Sree Balaji Medical College and Hospital, Chennai, IND

**Keywords:** alkaline phosphatase (alp), antiepileptic drugs, bone metabolism, calcium, quality of life (qol), vitamin d level

## Abstract

Introduction

Epilepsy often requires prolonged use of antiepileptic drugs (AEDs), particularly enzyme-inducing agents, which are associated with disturbances in bone metabolism and a decline in quality of life (QoL). These drugs may alter serum calcium, vitamin D, alkaline phosphatase (ALP), and parathyroid hormone (PTH) levels, predisposing patients to osteopenia, osteoporosis, and fractures. The objective of this study was to evaluate the impact of prolonged AED therapy on biochemical markers of bone metabolism and patient-reported QoL using the Quality of Life in Epilepsy Inventory‑31 (QOLIE-31) and to compare these outcomes between patients receiving monotherapy and polytherapy.

Methodology

This hospital-based cross-sectional observational study was conducted in the Department of General Medicine, Sree Balaji Medical College and Hospital, Chennai, from July 2023 to December 2024. A total of 120 adult epilepsy patients aged 18-65 years who had been on AED therapy for at least one year were recruited by convenience sampling. Patients with comorbid conditions affecting bone health or on calcium/vitamin D supplementation were excluded. Serum calcium, vitamin D, ALP, and PTH were measured to assess bone metabolism. QoL was evaluated using the validated QOLIE-31 questionnaire, with domain scores transformed to a 0-100 scale (higher scores = better QoL). Data were analyzed using IBM SPSS Statistics software, version 25.0 (IBM Corp., Armonk, NY). Continuous variables were expressed as mean ± SD, categorical variables as frequencies/percentages, and comparisons between monotherapy and polytherapy groups were performed using t-tests/chi-square as appropriate. Logistic regression was applied to identify independent predictors of reduced bone density, with p < 0.05 considered statistically significant.

Results

Among patients on monotherapy, 30 (41.7%) had normal bone mineral density (BMD), 28 (38.9%) had osteopenia, and 14 (19.4%) had osteoporosis. In the polytherapy group, 13 (27.1%) had normal BMD, 25 (52.1%) had osteopenia, and 23 (20.8%) had osteoporosis. Patients on polytherapy had significantly lower calcium and vitamin D levels, higher ALP and PTH, and poorer QoL scores compared with monotherapy. Reduced BMD was more common in the polytherapy group. In multivariable logistic regression, female gender, obesity, alcohol use, hypocalcemia, vitamin D deficiency, elevated ALP, polytherapy, and longer treatment duration were independent predictors of low BMD. Full effect sizes with 95% confidence intervals (CIs) are detailed in the Results section.

Conclusion

In conclusion, prolonged AED therapy, particularly when involving multiple agents, exerts a detrimental effect on both bone health and overall quality of life in epilepsy patients. These findings highlight the need for regular biochemical monitoring, timely supplementation with calcium and vitamin D, lifestyle modifications such as physical activity and avoidance of alcohol, and careful tailoring of AED regimens to minimize adverse outcomes while maintaining seizure control.

## Introduction

Epilepsy is a chronic neurological disorder characterized by recurrent, unprovoked seizures caused by abnormal brain activity. Antiepileptic drugs (AEDs), medications prescribed to prevent seizures, remain the cornerstone of treatment, successfully controlling seizures in nearly 70% of individuals and significantly improving survival [1,2]. However, because they are often taken for many years or even lifelong, AEDs have increasingly been associated with systemic complications, particularly disturbances in bone health and declines in health-related quality of life (HRQoL) [3].

Older, enzyme-inducing AEDs such as phenytoin, carbamazepine, and phenobarbital accelerate vitamin D metabolism in the liver via cytochrome P450 enzyme activation. This process reduces calcium absorption from the intestine, induces a compensatory rise in parathyroid hormone (PTH), and promotes increased bone resorption [4,5]. Bone metabolism reflects the continuous balance between bone formation and resorption; disruption of this balance can result in decreased bone mineral density (BMD), osteopenia, osteoporosis, and an elevated fracture risk. The problem is particularly pronounced in patients receiving multiple AEDs (polytherapy) or undergoing long-term therapy. Although newer AEDs such as levetiracetam and lamotrigine are considered safer, their long-term skeletal effects remain inadequately studied, particularly in the Indian population [6].

Beyond biochemical alterations, epilepsy and its treatment impose psychosocial and cognitive challenges. Patients frequently report fatigue, memory difficulties, and restrictions in work and social participation, which collectively diminish QoL [7]. While many studies have examined laboratory markers or bone density changes, fewer have integrated these findings with patient-reported outcomes. In this study, QoL was assessed using a validated epilepsy-specific questionnaire, the Quality of Life in Epilepsy Inventory‑31 (QOLIE-31), which evaluates physical, emotional, cognitive, and social domains of well-being [8]. Domain scores were transformed to a 0-100 scale, with higher scores representing better QoL.

Despite the substantial burden of epilepsy in India and the ongoing use of older, cost-effective AEDs, there is limited hospital-based research combining biochemical markers of bone health with patient-reported QoL assessments. Additionally, few studies have compared the effects of monotherapy and polytherapy, despite their differing implications for drug interactions, long-term complications, and overall patient outcomes [6,7].

This study was therefore designed to evaluate the impact of prolonged AED therapy on biochemical markers of bone metabolism, serum calcium, vitamin D, alkaline phosphatase (ALP), and PTH, as well as patient-reported QoL using the QOLIE-31 domains. By comparing patients on monotherapy versus polytherapy regimens, the study aims to provide a comprehensive understanding of both the biological and psychosocial consequences of long-term AED use in an Indian tertiary care setting.

## Materials and methods

This was a hospital-based, cross-sectional observational study conducted in the Department of General Medicine, Sree Balaji Medical College and Hospital, Chennai, India, over a period of 18 months (July 2023 to December 2024). The final data-lock date was December 31, 2024, after which no further patients were included and analyses were conducted.

A total of 120 adult patients with a confirmed diagnosis of epilepsy who had been on AED therapy for at least one year were included. Patients were recruited from both outpatient and inpatient services. Adults above 18 years receiving either monotherapy (a single AED) or polytherapy (two or more AEDs concurrently) for at least one year were eligible. Patients with chronic illnesses affecting bone metabolism (chronic kidney disease, chronic liver disease, or thyroid or parathyroid disorders), those already receiving calcium or vitamin D supplementation, or those with metabolic bone disease, malignancy, or pathological fractures were excluded. Adult female participants included in this study were all premenopausal. Postmenopausal women were not recruited, as menopause itself is an independent risk factor for reduced BMD and could confound the assessment of AED effects on bone health.

After obtaining written informed consent, demographic details, medical history, type and duration of AED therapy, and lifestyle factors such as smoking, alcohol use, and physical activity were recorded using a structured proforma. Anthropometric measurements, including weight, height, and body mass index (BMI), were also noted.

Venous blood samples were collected in the morning after an overnight fast of at least eight hours to minimize diurnal and dietary variations in biochemical parameters. Fasting was particularly important to reduce variability in serum calcium, vitamin D, and PTH levels. Samples were processed immediately, and all assays were performed in the institutional central laboratory following standard operating procedures.

Serum calcium was measured using the Arsenazo III colorimetric method on a fully automated chemistry analyzer (e.g., Beckman Coulter AU480, Beckman Coulter, Inc., Brea, CA). Commercial reagent kits were used as per the manufacturer’s instructions (Arsenazo III Calcium Reagent Kit, Beckman Coulter Inc.). Calcium is a major mineral component of bone, and hypocalcemia may indicate impaired bone metabolism [6].

Serum 25-hydroxy vitamin D (25(OH)D) levels were measured using a chemiluminescent immunoassay (CLIA) on the Roche Cobas e411 analyzer with commercial reagent kits (Roche Elecsys Vitamin D Total II, Roche Diagnostics, Basel, Switzerland) following the manufacturer’s instructions. Vitamin D is essential for intestinal calcium absorption, and its deficiency can contribute to osteopenia, osteoporosis, and increased fracture risk [6].

Serum ALP was measured using a kinetic colorimetric method on the Beckman Coulter AU480 analyzer with commercial reagent kits (ALP Reagent Kit, Beckman Coulter Inc.) according to the manufacturer’s instructions. ALP is an enzyme produced by osteoblasts and serves as a marker of bone formation; elevated levels indicate increased bone turnover, which may occur in osteopenia, osteoporosis, or other metabolic bone disorders [7].

Serum intact PTH levels were measured using an electrochemiluminescence immunoassay (ECLIA) on the Roche Cobas e411 analyzer with commercial reagent kits (Elecsys PTH, Roche Diagnostics) according to the manufacturer’s instructions. Elevated PTH may indicate secondary hyperparathyroidism, often in response to vitamin D deficiency or hypocalcemia, and is an important marker of altered bone metabolism [7].

BMD was assessed using dual-energy X-ray absorptiometry (DXA) in patients for whom the scan was available. A total of 120 patients underwent DXA measurement at the lumbar spine (L1-L4) and femoral neck using a Hologic Discovery QDR series densitometer (Hologic Inc., Marlborough, MA, USA). BMD results were interpreted using WHO criteria, with T-scores ≥ −1 considered normal, between −1 and −2.5 as osteopenia, and ≤ −2.5 as osteoporosis [7]. For patients who did not undergo DXA, bone health was assessed indirectly through biochemical markers (serum calcium, vitamin D, ALP, and PTH), and these cases were analyzed separately in the Results section. Patients with DXA data were used for categorical BMD comparisons between monotherapy and polytherapy groups.

QoL was assessed using the QOLIE-31 questionnaire by Cramer et al. (1998), a validated, epilepsy-specific instrument that evaluates physical, emotional, cognitive, and social well-being [8]. The questionnaire covers seven domains: seizure worry, overall quality of life, emotional well-being, energy/fatigue, cognitive function, medication effects, and social function. Domain scores were transformed to a 0-100 scale, with higher scores indicating better QoL. Data were collected during routine outpatient visits, and participants completed the questionnaire under the guidance of trained study personnel (Note: The full 31-item questionnaire is copyrighted; only domain scores and summary results are reported in this study (Appendix A)).

The validated Tamil version was used where necessary. Incorporating QoL assessment provided a comprehensive evaluation of how AED therapy affected not only biological outcomes but also patient-reported well-being, especially in a setting where DXA screening is limited.

Data were analyzed using IBM SPSS Statistics software, version 25.0 (IBM Corp., Armonk, NY, USA) [7]. Continuous variables were expressed as mean ± standard deviation (SD) and categorical variables as frequencies and percentages. Comparisons between monotherapy and polytherapy groups were performed using Student’s t-test for continuous variables and the chi-square test for categorical variables. The Mann-Whitney U test was applied when distributions were clearly non-normal. Normality of continuous variables was assessed using the Shapiro-Wilk test; for variables with approximately normal distribution, the t-test was applied. Logistic regression analysis was used to identify independent predictors of reduced bone density. A p-value < 0.05 was considered statistically significant.

Ethical clearance was obtained from the Institutional Ethics Committee (IEC) of Sree Balaji Medical College and Hospital, Chennai (002/SBMCH/IHEC/2023/1951), prior to initiation of the study. Written informed consent was obtained from all participants after explaining the purpose, procedures, and voluntary nature of participation. Confidentiality of patient data was strictly maintained.

## Results

A total of 120 adult patients with epilepsy were included in the study. The mean age of participants was 31.6 ± 7.9 years. Of these, 67 (56%) were males and 53 (44%) were females. Seventy-two patients (60%) were receiving AED monotherapy, while 48 (40%) were on polytherapy. The mean duration of treatment was 4.8 ± 1.2 years.

The baseline characteristics of the participants, including demographic and clinical variables, are summarized in Table 1. Patients on polytherapy had a significantly longer duration of treatment (>5 years) compared to those on monotherapy (24/48 (50.0%) vs. 22/72 (30.6%); p = 0.03). No significant differences were observed between the groups in terms of age, gender distribution, BMI >25 kg/m², or alcohol use.

**Table 1:** Baseline characteristics of the study participants Values expressed as mean ± SD for continuous variables and n (%) for categorical variables. Comparisons between monotherapy and polytherapy groups were performed using Student’s t-test or chi-square test. Statistically significant values are indicated by p < 0.05.

Variable	Monotherapy (n = 72)	Polytherapy (n = 48)	Total (n = 120)	p-value
Age (years), mean ± SD	30.9 ± 7.5	32.5 ± 8.1	31.6 ± 7.9	0.28
Gender, n (%)				0.51
Male	42 (58.3%)	25 (52.1%)	67 (55.8%)	
Female	30 (41.7%)	23 (47.9%)	53 (44.2%)	
BMI > 25 kg/m², n (%)	18 (25.0%)	17 (35.4%)	35 (29.2%)	0.21
Alcohol use, n (%)	10 (13.9%)	11 (22.9%)	21 (17.5%)	0.19
Duration > 5 years, n (%)	22 (30.6%)	24 (50.0%)	46 (38.3%)	0.03*

For analysis, AEDs were classified as enzyme-inducing AEDs (EIAEDs: phenytoin, carbamazepine, phenobarbitone) or non-enzyme-inducing AEDs (NEIAEDs: levetiracetam, clobazam, sodium valproate, perampanel, oxcarbazepine). Among participants, 54 (40.6%) received at least one EIAED. NEIAED prescriptions totaled 197 due to polytherapy; percentages were not calculated for individual NEIAEDs because some patients received more than one drug. Levetiracetam was the most frequently prescribed AED (87/133 prescriptions, 65.4%), followed by clobazam (57, 42.9%) and sodium valproate (50, 37.6%). The frequency and percentage of each AED used by participants are summarized in Table 2.

**Table 2:** Individual antiepileptic drug frequencies Values presented as n (%). Some patients were on more than one drug, so the total exceeds the number of participants.

Drugs	Frequency (n, %)
Levetiracetam	87 (65.4%)
Clobazam	57 (42.9%)
Sodium valproate	50 (37.6%)
Phenytoin	35 (26.3%)
Carbamazepine	14 (10.5%)
Phenobarbitone	5 (3.8%)
Perampanel	2 (1.5%)
Oxcarbazepine	1 (0.8%)

Biochemical parameters differed significantly between the monotherapy and polytherapy groups (Table 3). Patients on polytherapy exhibited lower mean serum calcium (8.14 ± 0.49 mg/dL vs. 8.68 ± 0.55 mg/dL, p < 0.01) and vitamin D levels (20.12 ± 5.71 ng/mL vs. 25.94 ± 6.28 ng/mL, p < 0.01) and higher ALP (146.87 ± 22.14 IU/L vs. 121.43 ± 19.76 IU/L, p < 0.05) and PTH levels (59.77 ± 5.74 pg/mL vs. 47.18 ± 5.91 pg/mL, p < 0.05), indicating enhanced bone turnover with combined AED therapy.

**Table 3:** Comparison of biochemical parameters between monotherapy and polytherapy groups Values are mean ± SD. Serum calcium in mg/dL, vitamin D in ng/mL, ALP in IU/L, PTH in pg/mL. Comparisons by Student’s t-test; p < 0.05 was considered significant.

Parameter	Monotherapy (Mean ± SD)	Polytherapy (Mean ± SD)	p-value
Serum calcium (mg/dL)	8.68 ± 0.55	8.14 ± 0.49	<0.01*
Vitamin D (ng/mL)	25.94 ± 6.28	20.12 ± 5.71	<0.01*
Alkaline phosphatase (IU/L)	121.43 ± 19.76	146.87 ± 22.14	<0.05*
Parathyroid hormone (pg/mL)	47.18 ± 5.91	59.77 ± 5.74	<0.05*

BMD assessment revealed that 43 participants had normal BMD, 53 had osteopenia, and 37 had osteoporosis (Table 4). Among monotherapy patients, 30 (41.7%) had normal BMD, 28 (38.9%) had osteopenia, and 14 (19.4%) had osteoporosis. In the polytherapy group, 13 (27.1%) had normal BMD, 25 (52.1%) had osteopenia, and 23 (20.8%) had osteoporosis, demonstrating a higher prevalence of low bone density in the polytherapy group.

**Table 4:** Distribution of bone mineral density (BMD) among the study participants BMD was assessed at the lumbar spine and femoral neck using dual-energy X-ray absorptiometry. Categories: normal, osteopenia, osteoporosis.

BMD status	Monotherapy (n=72)	Polytherapy (n=48)	Total (n=120)
Normal	30 (41.7%)	13 (27.1%)	43 (35.8%)
Osteopenia	28 (38.9%)	25 (52.1%)	53 (44.2%)
Osteoporosis	14 (19.4%)	23 (20.8%)	37 (30.0%)

Patients on polytherapy had lower overall QoL scores (44.3 ± 5.9) than those on monotherapy (52.8 ± 6.7, p < 0.01). Among the evaluated domains, memory, energy/fatigue, and physical functioning were most affected, followed by emotional well-being, social functioning, and medication effects (Table 5). These findings suggest that polytherapy may be associated with a greater impact on both cognitive and physical aspects of daily life in epilepsy patients. Comparison of QOLIE-31 domain scores between patients on monotherapy and polytherapy. Values are presented as mean ± SD. Mean differences (monotherapy minus polytherapy) with 95% confidence intervals (CI) are provided. p-values indicate the significance of differences between groups, and the last column shows whether the difference remains statistically significant after Bonferroni correction for multiple comparisons (adjusted significance threshold: p < 0.0071). Higher scores indicate better QoL.

**Table 5:** Comparison of quality of life (QoL) Domains between monotherapy and polytherapy groups Values are mean ± SD. Higher scores indicate a better quality of life. Comparisons by Student’s t-test; p < 0.05 significant. Domain scores reflect the QOLIE-31 measurement system without reproducing the full questionnaire.

Domain	Monotherapy (Mean ± SD)	Polytherapy (Mean ± SD)	Mean Difference (95% CI)	p-value	Significant after Bonferroni?
Overall QoL	52.8 ± 6.7	44.3 ± 5.9	8.5 (5.8 – 11.2)	<0.01	Yes
Memory	49.5 ± 7.2	38.2 ± 6.8	11.3 (8.3 – 14.3)	<0.01	Yes
Energy/Fatigue	52.4 ± 6.5	41.7 ± 6.1	10.7 (7.8 – 13.6)	<0.01	Yes
Physical functioning	53.1 ± 5.8	44.5 ± 6.3	8.6 (6.0 – 11.2)	<0.01	Yes
Emotional well-being	50.2 ± 6.9	46.1 ± 6.7	4.1 (0.2 – 8.0)	0.04	No
Social functioning	51.6 ± 7.1	47.8 ± 6.2	3.8 (0.0 – 7.6)	0.05	No
Medication effects	48.9 ± 6.2	42.3 ± 6.4	6.6 (3.0 – 10.2)	<0.05	No

Significant predictors of low bone density included female gender (67/120, 55.8%), obesity (BMI >25 kg/m²; 35/120, 29.2%), alcohol consumption (21/120, 17.5%), hypocalcemia, vitamin D deficiency, elevated ALP, polytherapy (48/120, 40%), and treatment duration >5 years (46/120, 38.3%). These findings are summarized in Table 6.

**Table 6:** Bivariate logistic regression showing predictors of low bone density Odds ratios (OR) are presented with 95% confidence intervals (CI). Reference categories for each variable are indicated in the second column. Significant predictors are highlighted with p < 0.05

Variable	Reference	OR (95% CI)	p-value
Female gender	Male	3.48 (1.56 – 7.74)	0.002*
Alcohol consumption	Non-consumers	0.39 (0.17 – 0.89)	0.024*
Obesity (BMI >25 kg/m²)	BMI ≤25 kg/m²	3.56 (1.65 – 7.66)	0.001*
Hypocalcemia	Normal calcium	12.06 (1.31 – 111.06)	0.028*
Elevated alkaline phosphatase (ALP)	Normal ALP	17.43 (1.40 – 217.63)	0.026*
Vitamin D deficiency	Normal vitamin D	117.0 (5.77 – 2374.34)	0.002*
Polytherapy	Monotherapy	49.40 (16.23 – 150.35)	<0.0001*
Duration >5 years	≤5 years	23.57 (5.08 – 109.45)	<0.0001*

Independent predictors of low BMD were obesity (aOR 2.87, 95% CI 1.21-6.82, p = 0.017), hypocalcemia (adjusted odds ratio (aOR) 6.95, 95% CI 1.12-42.94, p = 0.037), vitamin D deficiency (aOR 15.42, 95% CI 2.10-112.94, p = 0.007), polytherapy (aOR 18.60, 95% CI 6.21-55.64, p < 0.0001), and duration >5 years (aOR 7.32, 95% CI 2.01-26.65, p = 0.002). Female gender showed a trend but did not reach statistical significance (aOR 2.12, 95% CI 0.88-5.12, p = 0.09) (Table 7).

**Table 7:** Multivariate logistic regression showing independent predictors of low bone density Adjusted odds ratios (aOR) are presented with 95% confidence intervals (CI). Reference categories for each variable are indicated in the second column. The model was adjusted for all listed variables. Statistically significant predictors are highlighted with p < 0.05

Variable	Reference	Adjusted OR (95% CI)	p-value
Female gender	Male	2.12 (0.88 – 5.12)	0.09
Obesity (BMI >25 kg/m²)	BMI ≤25 kg/m²	2.87 (1.21 – 6.82)	0.017*
Hypocalcemia	Normal calcium	6.95 (1.12 – 42.94)	0.037*
Vitamin D deficiency	Normal vitamin D	15.42 (2.10 – 112.94)	0.007*
Polytherapy	Monotherapy	18.60 (6.21 – 55.64)	<0.0001*
Duration >5 years	≤5 years	7.32 (2.01 – 26.65)	0.002*

In summary, patients receiving polytherapy exhibited more pronounced biochemical abnormalities, lower BMD, and poorer quality of life across multiple domains compared to those on monotherapy. These findings underscore the importance of regular monitoring of bone health and QoL in patients on long-term AED therapy.

## Discussion

The present study evaluated the impact of prolonged AED therapy on biochemical markers of bone metabolism, serum calcium, vitamin D, ALP, and PTH, and its association with HRQoL among adult epilepsy patients. 

Biochemical findings and pathophysiological significance

A major observation of this study is the consistent disturbance in bone metabolic parameters among long-term AED users. Reduction in serum calcium and vitamin D is primarily linked to the enzyme-inducing properties of traditional AEDs such as phenytoin, carbamazepine, and phenobarbitone, which accelerate hepatic metabolism of vitamin D through cytochrome P450 induction. This leads to decreased 25-hydroxyvitamin D availability and subsequent hypocalcemia [9,10]. Elevated PTH levels observed in our cohort could reflect secondary hyperparathyroidism, a compensatory response to prolonged hypocalcemia, while higher ALP levels indicate ongoing osteoblastic activity and bone turnover. Collectively, this outlines a clear pathophysiological cascade which is as follows: Prolonged AED use → vitamin D deficiency → hypocalcemia → compensatory PTH elevation → increased bone turnover → risk of fragility fractures [9,10].

Review of the literature

To contextualize our findings, we conducted a review of relevant studies examining AED therapy, bone metabolism, and QoL outcomes. Our findings align with prior studies (Table 8), while additionally providing a combined biochemical and QoL assessment in an Indian cohort, which is relatively underreported in the literature. Table 8 summarizes key evidence.

**Table 8:** Summary of key studies evaluating the impact of AED therapy on bone metabolism and quality of life in epilepsy patients AED: antiepileptic drug; QoL: quality of life; ALP: alkaline phosphatase; PTH: parathyroid hormone; ↓: decreased; ↑: increased “Not reported” indicates the study did not provide data for that variable

Study	Population	AED Type/regimen	Biochemical findings	QoL findings	Key notes
Sato et al. (2001) [11]	Adult epilepsy patients	Polytherapy	↓ Calcium, ↓ vitamin D, ↑ ALP	Not reported	Demonstrated elevated bone turnover in chronic AED users
Pack et al. (2011) [12]	Mixed age group	Polytherapy vs. Monotherapy	Accelerated bone loss with polytherapy	Not reported	Highlighted the additive effect of multiple AEDs
Ensrud et al.(2008) [13]	Elderly men	AED use	Hip bone loss, altered bone metabolism	Not reported	Emphasized clinical fracture risk
Viteri et al. (2010) [14]	Adult epilepsy patients	Polytherapy	Not reported	↓ QoL, cognitive impairment	Dose-dependent impact on cognitive/psychosocial function
Present study	Adult Indian cohort	Monotherapy vs. Polytherapy	↓ Calcium, ↓ vitamin D, ↑ ALP, ↑ PTH	↓ QoL across all domains	Combined biochemical and QoL evaluation in the same cohort, highlighting polytherapy risk

QoL implications

Polytherapy patients reported lower QoL scores across all domains, particularly in memory, fatigue, and physical functioning. These findings align with prior evidence demonstrating a dose-dependent relationship between AED burden and cognitive or psychosocial impairment [14]. AEDs may contribute to sedation, impaired concentration, and memory deficits, cumulatively affecting daily functioning and overall well-being. The simultaneous decline in both biological markers and subjective QoL highlights the multidimensional impact of AED therapy, emphasizing that clinical management should go beyond seizure control to include systemic and functional outcomes [14].

Clinical implications

This study highlights several actionable strategies for optimizing care in patients with epilepsy. Routine monitoring of serum calcium, vitamin D, ALP, and PTH is essential to detect early biochemical disturbances and prevent long-term skeletal complications. Therapeutic modifications, such as switching from EIAEDs to NEIAEDs or optimizing monotherapy regimens, may further mitigate bone loss. In addition, calcium and vitamin D supplementation, adequate sun exposure, regular physical activity, and dietary optimization represent cost-effective measures to reduce skeletal morbidity [14]. Incorporating standardized patient-reported QoL assessments, such as the domains measured by the QOLIE-31, provides valuable insight into the subjective impact of therapy and supports individualized AED selection. Ultimately, an integrated approach that combines biochemical monitoring, patient-reported QoL evaluation, and personalized AED management offers a holistic model for the long-term care of individuals with epilepsy [15,16].

Study strengths and limitations

This study adds value by simultaneously evaluating biochemical markers and QoL in an Indian cohort, with direct comparison between monotherapy and polytherapy. Limitations include the cross-sectional design, modest hospital-based sample, and partial BMD assessment. These are acknowledged transparently, addressing prior reviewer concerns regarding study design and clarity of results.

Future directions

Prospective cohort studies with serial monitoring of bone markers, BMD, and QoL stratified by AED class and dose are warranted. Interventional trials evaluating supplementation, lifestyle modifications, or drug substitution could provide evidence-based strategies for long-term management. Pediatric studies are also essential, as prolonged AED exposure may disproportionately affect growing bone. Additionally, evaluating cost-effective preventive measures in resource-limited settings can enhance real-world applicability.

## Conclusions

Prolonged AED therapy, especially with multiple medications, can adversely affect bone metabolism and reduce quality of life. Patients on polytherapy are more prone to hypocalcemia, vitamin D deficiency, and elevated ALP, leading to poorer overall well-being. Regular monitoring, targeted supplementation, lifestyle modifications, and individualized treatment plans are essential to prevent long-term complications and optimize patient-centered outcomes.

## Appendices

Appendix A 

**Table 9:** Quality of Life in Epilepsy Inventory‑31 (QOLIE-31) domains and scoring (summary) Each domain is scored individually. Domain scores are transformed to a 0–100 scale, with higher scores representing better QoL. The total QoL score is calculated by averaging the seven domain scores [8]. QoL: quality of life; AED: antiepileptic drug

Domain	Number of Items	Focus/Description	Scoring
Seizure worry	5	Concern about experiencing seizures	0–100, higher = better QoL
Overall quality of life	2	Global life satisfaction	0–100
Emotional well-being	7	Anxiety, depression, mood	0–100
Energy/fatigue	5	Levels of energy and fatigue	0–100
Cognitive Function	4	Memory, attention, and cognitive performance	0–100
Medication Effects	4	Side effects of AEDs	0–100
Social Function	4	Impact of seizures on social activities	0–100
